# The complete mitochondrial genome of the critically endangered Lesser Antillean iguana (*Iguana delicatissima*; Squamata: Iguanidae)

**DOI:** 10.1080/23802359.2019.1637789

**Published:** 2019-07-12

**Authors:** Aryeh H. Miller, Anna C. Jackson, Matthijs P. van den Burg, Charles R. Knapp, Mark E. Welch, R. Graham Reynolds

**Affiliations:** a Department of Biology, University of North Carolina Asheville, Asheville, NC, USA;; b Department of Vertebrate Zoology, Smithsonian Institution, National Museum of Natural History, Washington, DC, USA;; c Department of Biological Sciences, Mississippi State University, Starkville, MS, USA;; d Institute of Biodiversity and Ecosystem Dynamics (IBED), University of Amsterdam, Amsterdam, The Netherlands;; e Department of Biogeography and Global Change, Museo Nacional de Ciencias Naturales (MNCN), Spanish National Research Council (CSIC), Madrid, Spain;; f Daniel P. Haerther Center for Conservation and Research, John G. Shedd Aquarium, Chicago, IL, USA

**Keywords:** Caribbean, Illumina, mitogenome, next-generation sequencing, phylogeny

## Abstract

The Lesser Antillean iguana, *Iguana delicatissima* Laurenti 1768, is one of the most endangered vertebrate taxa in the West Indies. This species faces significant threats, including introgressive hybridization with the introduced congener *Iguana iguana*. We deploy a combination of off-target sequence capture obtained from Illumina^®^ reads and targeted Sanger reads to assemble the mitochondrial genome of *I. delicatissima*. The mitogenome is 16,616 bp in length and is comprised of 13 protein-coding genes, two ribosomal subunits (rRNAs), 22 transfer RNAs, and a control region. Gene order is identical to that of congener *I. iguana* and other closely related taxa, absent of any tandem repeat regions. We show the phylogenetic utility of the mitogenome with a maximum-likelihood analysis, which yields a topology concordant with previous studies of iguanine taxa. We are hopeful that this genomic resource will be useful in further informing applied conservation and management for this critically endangered species.

*Iguana delicatissima* is a critically endangered species endemic to the Lesser Antilles. Although originally on 14 islands, this species has experienced substantial population reduction and extirpation in the last century owing to harvesting, residential/commercial development, and agriculture (van den Burg et al. 2018a). The primary threat now is introgressive hybridization with introduced *I*. *iguana* (Knapp et al. 2014; Vuillaume et al. 2015; van den Burg et al. 2018b). Here we describe the first complete mitochondrial genome of *I. delicatissima* assembled using a combination of Illumina and Sanger technologies.

We sampled two adult *I. delicatissima* from wild populations from the Commonwealth of Dominica and Anguilla in 2008 (Martin et al. 2015), consisting of heparinized whole blood preserved in Longmire buffer stored at the Mississippi State University. We extracted whole genomic DNA and stored extracts at −20 °C. We then conducted Ultraconserved Elements sequence capture using the Tetrapods 5kv1 primer set followed by 150 base pair (bp) paired-end sequencing on an Illumina^®^ HiSeq 2500 run. We processed reads using the phyluce bioinformatics pipeline as detailed in prior studies (Faircloth et al. 2012; Faircloth 2013, 2016) to clean, contig, assemble, and align sequence reads. We mapped aligned loci to a reference (*I. iguana*; NC_002793; Janke et al. 2001) in Geneious 10.2.5 (Biomatters^®^). The consensus is chimeric, in that it is a consensus of nucleotides from two individuals from the same population; thus, there were 43 ambiguities retained in the final mitogenome sequence. We used the MITOS2 webserver (Bernt et al. 2013) to annotate the consensus sequence. We used custom-designed primers followed by Sanger sequencing to span regions with missing data. We then aligned the consensus sequence with other available iguanine mitogenomes available on GenBank using ClustalW 2.1 (Larkin et al. 2007) and conducted a maximum-likelihood analysis using RaxML (v8.2.9, Stamatakis 2014).

The complete mitochondrial genome of *I. delicatissima* (GenBank MK923980) is 16,616 bp in length, slightly shorter than that of congener *I. iguana* (16,633 bp). The overall nucleotide composition is 32.8% A, 31.3% C, 13.1% G, and 22.5% T. We found no rearrangements in the mitogenomic gene order of *I. delicatissima* in comparison to other closely related squamates, with 22 tRNAs, two ribosomal subunits, 13 protein-coding loci, an origin of light-strand replication (O_L_), and a control region. All tRNAs conformed to their stem and loop secondary structures, but we note that Trn-C had an incomplete D-arm. The O_L_ is 25 bp in length and is located in the WANCY tRNA cluster between tRNA-N and tRNA-C.

The phylogenetic analysis (Figure 1) yielded a topology congruent with previous studies of iguanine relationships. Sequence divergence among protein-coding genes in *I. delicatissima* and *I. iguana* is concordant with previous distance-based estimates between these two taxa (Stephen et al. 2013), with uncorrected pairwise divergences among protein-coding genes (p-distances) ranging from 9.0% (ND2) to 13.4% (ATP6). Characterizing non-traditional loci (e.g. whole mitogenomes) adds to a growing database of genomic resources for aiding conservation efforts and further elucidating the evolutionary history of this remarkable and endangered species.

**Figure 1.**
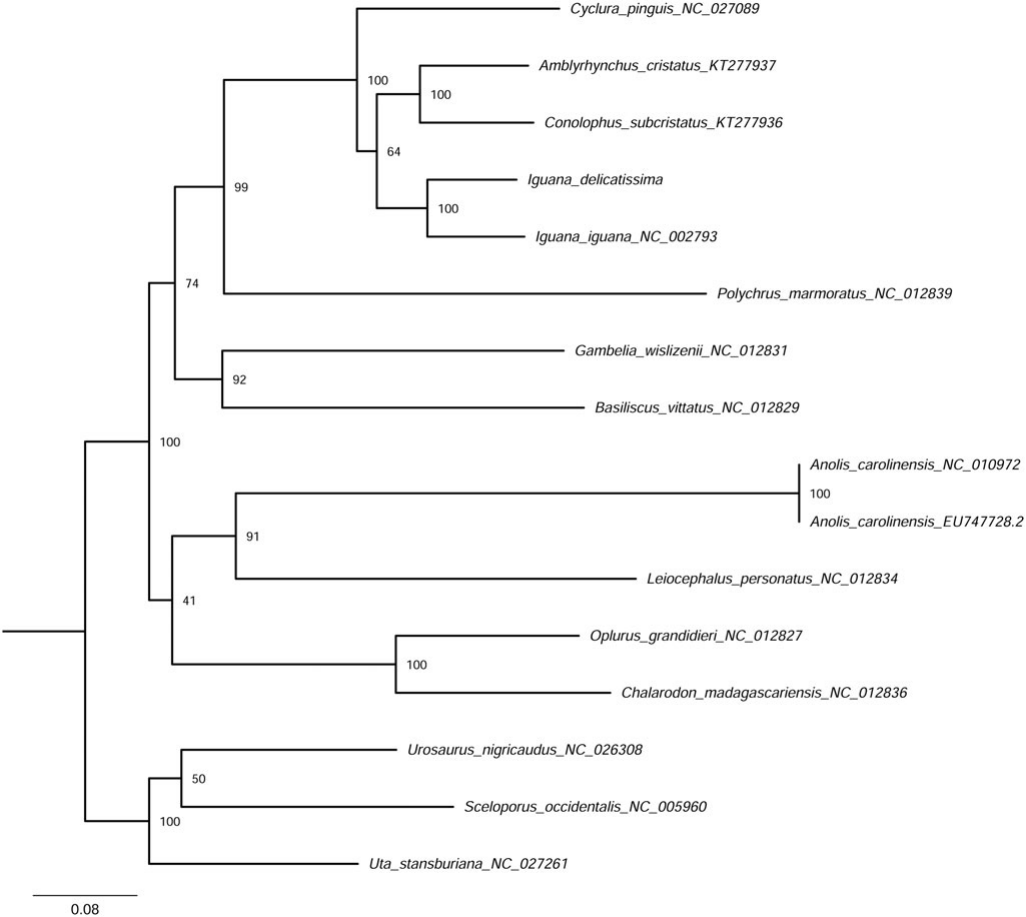
Maximum-likelihood phylogeny of aligned concatenated protein-coding loci (11,363 bp) with GenBank material utilizing the rapid bootstrap inferences (1000 replicates) followed by a thorough ML search option. Numbers at nodes represent bootstrap support.
